# Initial nucleation of nanodroplets in viscoelastic tissue driven by ultrasound: A theoretical simulation^☆^

**DOI:** 10.1016/j.ultsonch.2025.107285

**Published:** 2025-02-24

**Authors:** Kangyi Feng, Yueyuan Wang, Chaonan Zhang, Anqi Huang, Mingxi Wan, Yujin Zong

**Affiliations:** The Key Laboratory of Biomedical Information Engineering of Ministry of Education, School of Life Science and Technology, Xi’an Jiaotong University, Xi’an 710049, People's Republic of China

**Keywords:** Ultrasound, Phase-change nanodroplet, Nucleation threshold, Viscoelastic tissue

## Abstract

Phase-change nanodroplets hold promising potential for theranostic applications in tumor tissue. However, the initial nucleation of nanodroplets in tissue—a critical stage for subsequent vapor bubble dynamics and theranostic efficacy—remains unexplored. This work, accounting for nanodroplets and tissue as compressible mediums, was represented by two springs in series: one for nanodroplet compressibility and the other for tissue elasticity. By analyzing the linear relationship between internal nanodroplet pressure and volume changes in nanodroplets and tissue, the classical nucleation theory (CNT) was modified to describe the initial nucleation of perfluoropentane (PFP) nanodroplets in tissue. The key nucleation conditions, such as the stable critical radius and initial nucleation threshold (INT) were investigated based on the modified CNT. Results revealed that introducing the nanodroplet compressibility and tissue elasticity allows the existence of a stable critical radius—which is more physically meaningful, highlighting their important effects on nucleation. Additionally, the INT increased significantly with the increase in tissue bulk modulus. For example, with an increase in bulk modulus from 0.03 MPa to 0.67 MPa, the INT increased by about 1.1 MPa. The increased behavior was more obvious for smaller nanodroplets in higher bulk modulus. The presence of dissolved gases, increasing nanodroplet surface tension, and decreasing nanodroplet radius and ultrasound frequency reduced the INT. Further analysis of the achievable nucleation area in tissue, which was expanded significantly at lower frequencies. Overall, this study enhances the understanding of initial nanodroplet nucleation in tissue, offering insights into designing and optimizing nanodroplet-based theranostic strategies.

## 1 Introduction

Phase-change nanodroplets, typically consisting of a volatile liquid core surrounded by a stabilizing shell, have shown great potential in various theragnostic applications [1], [2]. Compared to conventional microbubbles restricted in the vasculature, their sub-micron size and extended circulation time in *vivo* enable selective extravasation into tumoral tissue *via* the enhanced permeability and retention (EPR) effect. They have relatively lower boiling points, sometimes lower than the physiological temperature, such as perfluoropentane (PFP, 29 ℃). Due to the encapsulated shell and the prominent Laplace pressure, the pressure inside nanodroplets can be more than the saturating vapor pressure, allowing them to remain metastable at physiological temperatures and prevent spontaneous evaporation. Upon ultrasonic simulation, they can be selectively vaporized into high-echo microbubbles in a specific area of interest, primarily in tumor tissue, thus enabling enhanced imaging contrast [3], [4], on-demand release of theranostic cargoes [5], [6], or targeted drug delivery [7], [8].

In recent years, a concentric 3-layer geometry model (vapor-droplet-water/tissue) has been widely used to investigate the vaporization process of the droplet and determine the vaporization threshold [9], [10], [11], [12]. Notably, the methodology relies on the assumption that a stable vapor bubble already nucleated spontaneously at some time before ultrasonic simulation and stable persisted in bodily fluids, allowing investigation of droplet vaporization by the vapor bubble growth. However, the vaporization process of the nanodroplet involves nucleation-growth transition mechanisms, and not all newly formed vapor bubbles that appear instantaneously can exist and grow stably [13]. Only the vapor bubble reaches one of the nucleation conditions such as critical radius (CR)—the size at which the combined bulk and surface free energies are maximized, which can exist stably. Below the critical radius, the vapor bubble is prone to dissolution due to Laplace pressure in the absence of a stabilizing force, which highlights the importance of the vapor bubble nucleation in the vaporization process.

The vapor bubble nucleation was assumed to occur outside the droplet (i.e., heterogeneous nucleation) according to recorded acoustic cavitation noise [14]. However, this assumption was refuted by the direct observations using ultra-high-speed microscopy, which demonstrated that vapor bubble nucleation is initiated by homogeneous nucleation within droplets [15], [16], [17]. One approach to investigating homogeneous nucleation is by using classical nucleation theory (CNT). Traditional CNT initially assumed that the surface tension of the vapor bubble equaled the macroscopic surface tension of a flat interface, also known as the ‘‘capillarity approximation’’, leading to significant errors in the estimating nucleation rate [18]. Nevertheless, by correcting the surface tension to an effective value, CNT has been effectively used to model nucleation in homogeneous media, such as water [19], [20], and biological fluids [21]. This approach has also been successfully applied to nano- [22] and micron-sized PFP droplets in water [23], [24]. For example, it was proposed that the effective surface tension of the vapor bubble could be expressed as a function of overpressure—the difference between vapor bubble pressure and the actual pressure within the nanodroplet—yielding higher nucleation rates and nucleation thresholds closer to experimental values [25]. Additional parameters have also been incorporated into CNT for improved modeling of vapor bubble nucleation. For instance, Miles *et al*. combined CNT with the effects of nonlinear ultrasonic pressure distortion to predict the nucleation threshold of PFP droplets based on variables such as droplet radius, ultrasonic frequency, and medium temperature [24]. More recently, the potential influence of non-condensable dissolved gases (e.g., O_2_, CO_2_) on vapor bubble nucleation in liquid PFP nanodroplets has been incorporated into the CNT [26]. These refinements and additional considerations in CNT help advance our understanding of the initial nucleation of PFP droplets.

However, most studies on PFP droplet-related nucleation have focused on modeling the initial nucleation of droplets in water for theranostic intravascular applications, often treating the liquid-phase PFP and/or water as incompressible media for simplicity. In such incompressible situations, vapor bubble nucleation is accommodated by pushing liquid away and by modifications of the nanodroplet radius. This approach may not adequately model nanodroplet nucleation in tissue, where the surrounding solid-phase tissue behaves as a compressible medium with inherent elasticity. In this compressible environment, both the volume and size of the nanodroplet and tissue may vary, accompanied by deformations in both the nanodroplet and the surrounding tissue. The compressibility of the nanodroplet and the elasticity of the tissue may be therefore critical factors in calculating nucleation conditions using CNT for nanodroplet nucleation in tissue. Previous numerical studies have demonstrated that tissue elasticity not only dampens vapor bubble growth but also increases the vaporization threshold of nanodroplets [10]. An experimental study revealed that the phase-change thresholds of droplets in polyacrylamide phantoms increased as the stiffness of the surrounding phantoms stiffness increased [27]. In addition to the vaporization threshold, other parameters such as maximum microbubble expansion [28] and the number of formed microbubbles [27] were also significantly influenced. These findings highlight the significant impact of the medium properties on the nanodroplet vaporization process. However, unknowns remain in understanding the initial nanodroplet nucleation in tissue, especially for key components such as critical radius and the required ultrasonic pressure for nucleation–initial nucleation threshold (INT), and the nucleation areas in tissue.

In this work, we used a similar concentric 3-layer (vapor-nanodroplet-tissue) model to investigate the initial nucleation of a nanodroplet in tissue by incorporating revised factors into the CNT, including the surface tension of the vapor bubble, the presence of non-condensable dissolved gas, nanodroplet compressibility, and tissue elasticity. By deriving the work required for the nucleation of a vapor bubble with respect to their radial deformations, we obtained key components in the implementation of CNT in tissue such as the critical radius of the vapor bubble, the nucleation rate, and the INT. Numerical simulations were performed to explore the effects of the nanodroplet compressibility and tissue elasticity on the critical radius. Additionally, the effects of tissue bulk modulus, the non-condensable dissolved gas, nanodroplet parameters, and ultrasonic frequency on the INT were analyzed. Furthermore, by combining the peak negative pressure distribution in the focal regions of the ultrasonic field with the predicted INT for nanodroplets in tissue, we estimated the achievable nucleation areas where vapor bubbles would first nucleate.

## 2 Theory and methods

**Fig. 1:**
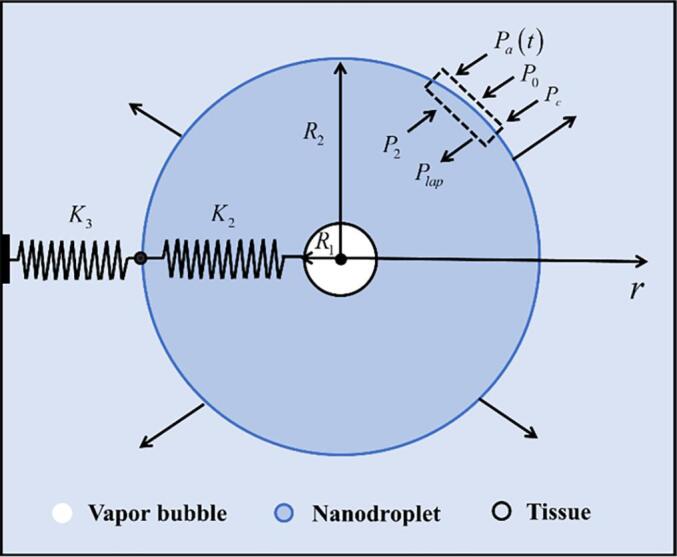
The schematic diagram for modeling the initial nucleation of a nanodroplet in tissue. Under ultrasonic simulation, nucleated a vapor bubble of radius *R*_1_ occurred in a PFP nanodroplet in tissue. During nucleation, the surrounding nanodroplet and tissue of the vapor bubble behave as springs mounted in series, with one formed by the nanodroplet compressibility of *K*_2_ and the other by the tissue elasticity of *K*_3_.

Fig. 1 illustrates a concentric 3-layer (vapor-nanodroplet-tissue) geometry model for the initial nucleation of a nanodroplet in tissue under ultrasonic fields. In this model, subscripts 1, 2, and 3 represent the vapor bubble, nanodroplet, and tissue, respectively. Upon ultrasonic simulation, a vapor bubble with a radius of *R*_1_ nucleated at the center of the nanodroplet with a radius of *R*_2_ in tissue. The nucleation of the vapor bubble is accompanied by the deformation in both the nanodroplet and the surrounding tissue. This process can be modeled as two springs in series, with one formed by the nanodroplet compressibility and the other by the tissue elasticity. The compressibility and elasticity were characterized by the isothermal bulk moduli *K*_2_ and the tissue *K*_3_, respectively. *P*_c_ is the constraint forces exerted on the nanodroplet surface by tissue elasticity.

### 2.1 Local pressure description in a nanodroplet surrounded by tissue

In the following analysis, we adopt the common assumption that pressure within the nanodroplet and vapor bubble is spatially uniform. This implies that the pressure on the inner wall of the surrounding liquid is approximately equal to the liquid pressure inside the nanodroplet. To simplify subsequent expressions and discussions, when disregarding nanodroplet compressibility and tissue elasticity we start by defining the local liquid pressure at the nanodroplet surface, as $P_{2w0}$:

(1) $$P_{2wo}=P_{0}+P_{\mathrm{lap}}+P_{a}\left(t\right),$$

where $P_{0}$, $P_{\mathrm{lap}}$, and $P_{a}\left(t\right)$ are the ambient pressure, Laplace pressure, and applied ultrasonic pressure, respectively. The Laplace pressure experienced by a nanodroplet is given by $P_{\mathrm{lap}}=\sigma_{2}/R_{20}$, where $\sigma_{2}$ is the surface tension at the liquid-tissue interface of the nanodroplet. According to the classic CNT, the CR in a Young-Laplace-type equation can be written as [29]:

(2) $$R_{1wo}^{*}=\frac{-2\sigma_{1}}{P_{2wo}-P_{1}},$$

where $\sigma_{1}$ is the surface tension of the vapor bubble at the liquid–vapor interface, and $P_{1}$ is the vapor pressure inside the vapor bubble.

To describe the effect of nanodroplet compressibility and tissue elasticity, it is essential to understand how the liquid pressure relates to the vapor bubble nucleation. We assumed that the volume of the vapor bubble, *V*_1_, is much smaller compared to the volume of the nanodroplet, *V*_2_, i.e., ${\left(R_{1}/R_{2}\right)}^{3}\ll 1$, and that the number of liquid molecules remains constant, neglecting any mass loss due to the vaporization or condensation [30]. As mentioned in the introduction, nucleating a vapor bubble is accompanied by the deformation of the liquid nanodroplet, with a focus on its volumetric deformations. Based on Eq. (1), the relation between the local liquid pressure, $P_{2}$, and the transient nanodroplet volume, $V_{2}$, follows the liquid equation of state and can be linearized in the form [31]:

(3) $$P_{2}=P_{2wo}-K_{2}\frac{V_{2}-V_{20}}{V_{20}},$$

where $V_{20}$ represents the volume of the nanodroplet at *t* = 0, when no vapor bubble exists at the initial moment and the liquid occupies the entire volume of the confinement, then having $V_{20}=4\pi {R_{20}}^{3}/3$.

Similarly, nucleating a vapor bubble at the center of the nanodroplet is also accompanied by the volumetric deformations of the tissue, i.e., $V_{3}\;>V_{20}$. Under the assumption of small-volume deformations, the linear relationship between the liquid pressure and the tissue volume thus can be written as [31]:

(4) $$P_{2}=P_{2wo}+K_{3}\frac{V_{3}-V_{20}}{V_{20}},$$

where $V_{3}$ is the transient volume of the tissue. Notably, the opposite sign of the bulk modulus of the tissue, compared to that of the nanodroplet (Eq. (3), ensures that the tissue effects remain positive. By combining Eqs. (3), (4), and using the relationship that the transient tissue volume is equal to the sum of the transient nanodroplet volume and the vapor bubble volume ($V_{3}=V_{2}+V_{1}$), can be obtained:

(5) $$P_{2}=P_{2wo}+K{\left(\frac{R_{1}^{*}}{R_{20}}\right)}^{3},$$

(6) $$K=\frac{K_{2}K_{3}}{K_{2}+K_{2}}.$$

where $R_{1}^{*}$ is the stable critical radius (SCR) of the vapor bubble during the nucleation process, which will be described in the following chapters. *K* represents the harmonic average of the nanodroplet modulus $K_{2}$ and of the tissue modulus $K_{3}$, which can be seen as two springs in series, with one formed by the nanodroplet compressibility and the other by the tissue elasticity.

### 2.2 Initial nucleation thresholds of a nanodroplet in tissue based on a modified CNT

Nucleation of a vapor bubble in a nanodroplet is an activated process that requires overcoming an energy barrier, which is influenced by the free energy at the liquid–vapor interface and the chemical potential energy. The free energy consists of a surface term, proportional to the surface area of the vapor bubble proportional to the surface area of the vapor bubble, and a bulk term, proportional to its volume. The work *W* required to nucleate a vapor bubble with a radius *R*_1_ in the central of the nanodroplet is given by [32]:

(7) $$W\left(R_{1}\right)=\left(4\pi R_{1}^{2}\right)\sigma_{1}+\left(\frac{4\pi R_{1}^{3}}{3}\right)\left(P_{2}-P_{1}\right)+N\left(\mu_{1}-\mu_{2}\right),$$

where *N* is the number of molecules inside the vapor bubble, and $\mu_{1}$ and $\mu_{2}$ are the chemical potentials of the vapor and liquid phases, respectively. The presence of dissolved gases can influence the vapor pressure inside the vapor bubble $P_{1}$, which can be expressed as [33]:

(8) $$P_{1}=P_{v1}+P_{g1},$$

where $P_{v1}$ is the vapor pressure of the liquid, and $P_{g1}$ is the partial pressure of the non-condensable dissolved gases. According to Henry’s law, the partial pressures of non-condensable dissolved gases in the vapor bubble can be written as [34]:

(9) $$P_{g1}=\sum_{i}K_{i}C_{\mathrm{gi}},$$

where $K_{i}$ and $C_{\mathrm{gi}}$ are the volatility constant and concentration of the *i*-th type of dissolved gas, respectively, in the liquid PFP. The dissolved gas CO_2_ and O_2_ were considered in the PFC nanodroplet, as their solubility shows a higher proportion in the non-condensable dissolved gases.

The size-dependent work increases until it reaches a maximum value of W* at $R_{1}^{*}$ and then goes to decrease. The vapor bubble becomes stable only when its radius is equal to or greater than *R*_1_*. This is mathematically expressed as${\left(dW/{\mathrm{dR}}_{1}\right|}_{R_{1}=R_{1}^{*}}$ = 0. At $R_{1}^{*}$, the vapor bubble is in a thermodynamic balance with the liquid nanodroplet, which implies chemical equilibrium *μ*_2_ = *μ*_1_. Thus, $R_{1}^{*}$ and the corresponding critical work $W^{*}$ can be derived using the following mathematical definitions:

(10) $$R_{1}^{*}=\frac{2\sigma_{1}}{P_{1}-P_{2}},$$

(11) $$W^{*}=\frac{16\pi }{3}\frac{\sigma_{1}^{3}}{{\left(P_{1}-P_{2}\right)}^{2}},$$

Accordingly, the nucleation rate—the number of vapor bubbles with a critical radius formed per unit of time and volume—is proportional to the difference between the growth and condensation rates when the vapor bubble reaches the critical radius. This rate can be considered stationary, as the negative pressure period is much more extended than the timescales required for nucleation. The nucleation rate is determined by the corresponding critical work $W^{*}$, which is usually written as:

(12) $$J=J_{0}\exp\left(\frac{-16\pi \sigma_{1}^{3}}{3k_{B}T_{0}{\left(P_{1}-P_{2}\right)}^{2}}\right),$$

where $J_{0}=\sqrt{3\sigma_{1}\rho_{2}^{2}/\pi m}$ is the kinetic pre-exponential factor, which accounts for the average kinetic and spatial characteristics of nucleation and represents the dimensionless energy required to form a critical vapor bubble [32]. *m* is the mass of the single molecule, and $\rho_{2}$ is the temperature-dependent density of the liquid nanodroplet, *T*_0_ is the initial temperature inside the liquid nanodroplet. A correct density of the liquid nanodroplet that is dependent on the temperature in a power series can be given by [35]:

(13) $$\rho_{2}=\rho_{20}\left[1+\sum_{i=1}^{6}B_{i}{\left(1-T_{0}/T_{c}\right)}^{i/3}\right],$$

where $\rho_{20}=759.53\;{kg/m}^{3}$ is the critical density of the PFP nanodroplet, $T_{c}=\;420\;K$ is the critical temperature of PFP nanodroplet, and the series of constants *B* are given as: $B_{1}=\;-0.425$; $B_{2}=\;8.191$; $B_{3}=\;-17.91$; $B_{4}=\;19.63$; $B_{5}=\;-10.92$; $B_{6}=2.655$.

For steady-state nucleation, the number of vapor bubbles $N_{b}$ formed within the volume of the liquid nanodroplet *V*_20_ during the time interval *τ* can be approximated as $N_{b}≅J\left(P_{2},T_{0}\right)V_{20}\tau$, where *τ* defines the time interval over which the first vapor bubble nucleates [29]. According to Eq. (12), the nucleation rate is time-independent, while the liquid pressure and temperature are transient. Under this condition, it can be assumed that the time interval is sufficiently short, so no significant changes in the nucleation rate occur due to variations in *P*_2_ and *T*_0_. Therefore, *τ* can be modeled as a fraction of the ultrasound wave, where the pressure values are the lowest and remain relatively constant, and it is approximated as τ=1/10f [29], where f is the ultrasound frequency. According to the CNT, the probability of the vapor bubble nucleation in the liquid nanodroplet is $\sum =1-exp\left(-JV_{20}\tau \right)$. The initial nucleation threshold, *P*_INT_, is defined as the absolute value of the ultrasonic pressure at which the nucleation probability reaches 50 %. Thus, submitting Eq. (1), Eq. (5), Eq. (8), and Eq. (12) into the probability of the vapor bubble nucleation, the *P*_INT_ is given by:

(14) $$P_{\text{INT}}=P_{v1}+P_{g1}-P_{0}-P_{\mathrm{lap}}-{\left(\frac{16\pi \sigma_{1}^{3}}{3k_{B}T_{0}\left(J_{0}V_{20}\tau /\ln2\right)}\right)}^{1/2}.$$

From Eqs. (10)-(12), it is evident that the required nucleation work and rate are closely related to the surface tension of the vapor bubble at the liquid–vapor interface. In our modified CNT, the surface tension of the vapor bubble is considered size-, pressure-, and temperature-dependent at nucleation. The revised surface tension, $\sigma_{1}$, is expressed as a function of the experimentally controllable scaled overpressure of the liquid phase, and is given by [36]:

(15) $$\sigma_{1}=\sigma_{\infty }\left(T_{0}\right)-\varphi^{1/3},$$

(16) $$\varphi =\left(1-\delta \right){\left(1+0.5\delta \right)}^{2},$$

where φ is determined by the actual liquid pressure *P*_2_, temperature *T*_0_, and the phase-change thermodynamics of the nanodroplet. An appropriate asymptotic relation for the temperature-dependent macroscopic surface tension is *σ*_∞_ (T_0_) = A(1-*T*_0_/*T*_c_)^2^*^v^*, where A is the constant coefficient, and *υ* is the critical exponent that describes the behavior of the surface tension as approaching the critical point. The values A=0.0425 and υ=0.6 were used to calculate the macroscopic surface tension. At the same temperature, *δ* is given by [37]:

(17) $$\delta =\frac{\Delta P}{\Delta P_{s}}=\frac{P_{\mathrm{sat}}\left(T_{0}\right)-P_{2}\left(T_{0}\right)}{P_{\mathrm{sat}}\left(T_{0}\right)-P_{\mathrm{spin}}\left(T_{0}\right)},$$

where *δ* is a measure of the degree of liquid metastability, *P*_sat_ and *P*_spin_ represent the saturation vapor pressure and spinodal pressure of the PFP nanodroplet, respectively. The spinodal pressure *P*_spin_ is defined as the point at which the second derivative of pressure with respect to volume becomes zero during nanodroplet nucleation, marking the transition from liquid to vapor.

The classic cubic Redlich–Kwong (R-K) equation estimates the phase-change thermodynamics of a nanodroplet transitioning from liquid to vapor. It provides an algebraic relationship between pressure (*P*), molar volume (*V*), and temperature (*T*). The R-K equation is widely utilized for its reliability and simplicity, and can be generally represented as [38]:

(18) $$P=\frac{\mathrm{RT}}{V-b}-\frac{a\alpha \left(T\right)}{V\left(V+\xi \right)},$$

where *R* is the universal gas constant, the parameters *a* and *b* are evaluated as [38]:

(19) $$a=\frac{\Omega_{a}R^{2}T_{\text{c}}^{2}}{P_{\text{c}}},b=\frac{\Omega_{a}RT_{c}}{P_{\text{c}}}.$$

where Ω_a_ = 0.42748, Ω_b_ = 0.08664, and *ξ* = b are the constants. *α*(T) = 1/(*T*_r_)^½^, in which *T*_r_ = *T*/*T*_c_ is the reduced temperature, *P*_c_ is the critical pressure of PFP (*P*_c_ = 2.045 MPa) [39].

### 2.3 Computational conditions

Unless otherwise indicated, we considered the nucleation of a vapor bubble in a PFP nanodroplet in tissue at ambient pressure *P*_0_ = 1 atm, temperature *T*_0_ = 310 K, and saturation vapor pressure *P*_sat_ = 135 kPa. The applied ultrasound frequency *f* was 5 MHz, and the selected nanodroplet radius *R*_20_ = 140 nm, the surface tension at the nanodroplet interface *σ*_2_ = 56 mN/m. The bulk modulus of the PFP nanodroplet was used similarly to water, *K*_2_ = 2.2 GPa [39]. Moreover, the volatility constant *K*_i_ is derived from Henry’s constant for PFC liquids and depends solely on the gas species and ambient temperature with minimal influence from pressure. The gas solubility in liquid PFC decreases in the following order: CO_2_ ≫ O_2_, the values of *K*_i_ is ${4.9\times 10}^{6}$ for O_2_ and ${7.2\times 10}^{5}$ for CO_2_, the values of *C*_gi_ is 0.07 for O_2_ and 0.3 for CO_2_, respectively [26].

**Fig. 2:**
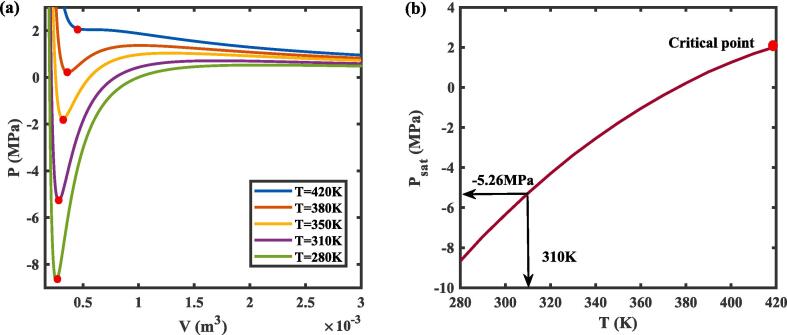
(a) Isotherms showing the relationship between pressure and volume at various temperatures, as determined by the Redlich-Kwong cubic equation. (b) The spinodal line, obtained by identifying the points where the second derivative of pressure with respect to volume equals zero, indicates the spinodal points.

To predict the INT of PFP nanodroplets, it is essential to determine the spinodal pressure of PFP, *P*_spin_. First, the pressure–volume isotherms for the PFP nanodroplet at various temperatures can be obtained by solving the classic cubic R–K equation (Eq.18), as shown in Fig. 2**a.** Below the critical temperature of the PFP ($T_{c}$ = 420 K), a local minimum is identified in the isotherm representing the liquid spinodal point. These spinodal points are determined where the second derivative of pressure with respect to volume equals zero [40]. By calculating the second derivative of the R-K equation at different temperatures, the points where this derivative is zero were plotted, forming the spinodal line, as shown in Fig. 2**b**. The spinodal pressure *P*_spin_ is equal to - 7.41 MPa at 294 K (21 ℃), - 6.32 MPa at 302 K (29℃), - 5.26 MPa at 310 K (37℃), and - 2.53 MPa at 343 K (70 ℃). These spinodal pressures will be used in the subsequent numerical simulations.

## 3 Results

### 3.1 Effect of nanodroplet compressibility and tissue elasticity on the critical radius of a vapor bubble

**Fig. 3:**
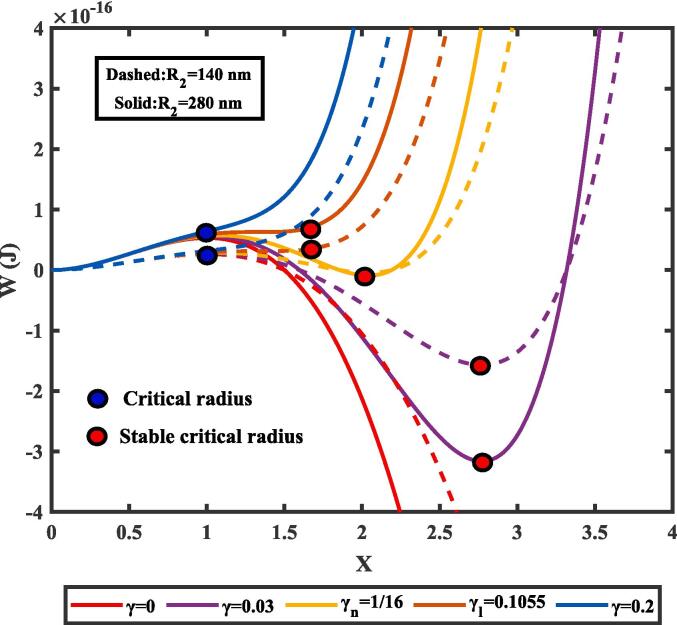
The nucleation work *W* as a function of the dimensionless radius *X* ,where ${X=R}_{1}/R_{1wo}^{*}$, was plotted for various values of the dimensionless parameters *γ*. The parameter *γ* represents the combined effects of nanodroplet compressibility and tissue elasticity on the initial nucleation of a nanodroplet in tissue. The critical radius $R_{1wo}^{*}$, and the stable critical radius $R_{1}^{*}$ were obtained from the classic nucleation theory and the modified-classic nucleation theory, respectively.

To emphasize the importance of nanodroplet compressibility and tissue elasticity, by submitting Eqs. (1)-(2) and (5) − (6) into Eq. (7), the work required for nucleation can be rewritten in a dimensionless form:

(20) $$\begin{array}{c} W\left(R_{1}\right)=W_{1wo}\left[3X^{2}-2X^{3}\left(1-\frac{\gamma }{4}\right)X^{3}\right]. \end{array}$$

where $W_{1wo}=4\pi R_{1wo}^{*}\sigma /3$ represents the typical energy barrier without considering the nanodroplet compressibility and tissue elasticity, ${X=R}_{1}/R_{1wo}^{*}$ denotes a dimensionless radius of the vapor bubble, and $\gamma =8\sigma^{3}K/R_{20}^{3}{\left(P_{2wo}-P_{1}\right)}^{4}$ is the dimensionless parameter that describes the effect of the nanodroplet compressibility and tissue elasticity. The work required for nucleation, *W*, as a function of the dimensionless radius, $R_{1}/R_{1wo}^{*}$, under different radii of the nanodroplet was shown in Fig. 3. The nucleation energy barrier dictates the lifetime of a metastable state of the nanodroplet, which can only persist until the spinodal point is reached. At or beyond the spinodal point, the energy barrier to nucleation disappears, allowing the phase change to occur spontaneously and often violently [40].

In Fig. 3, when *γ* = 0—that is, when nanodroplet compressibility and tissue elasticity were not considered—the vapor bubble first attained the critical radius (CR), $R_{1wo}^{*}$ (the blue circle in Fig. 3). Beyond this point, the vapor bubble experienced drastic, unbounded growth (reflected in the *W* continuous decrease). This unphysical phenomenon of infinite growth is well known and forms the basis of the unmodified CNT (*γ* = 0) traditionally used to describe cavitation and boiling [41]. However, when the effects of nanodroplet compressibility and tissue elasticity (*γ* ≠ 0) were taken into account, the vapor bubble did not undergo drastic, unbounded growth after reaching the CR. Instead, it underwent limited growth before stabilizing at a new equilibrium radius, referred to as the stable critical radius (SCR), $R_{1}^{*}$ (the red circle in Fig. 3). This stabilization arises from the combined effects of nanodroplet compressibility and tissue elasticity, which act to constrain vapor bubble growth and prevent the unbounded expansion typically observed in idealized models assuming incompressible media.

Furthermore, as the dimensionless parameter *γ* increased, the SCR gradually approached the CR, until the SCR disappeared at a limiting value, $\gamma_{l}$. This limit value can be determined by solving the conditions $\mathrm{dW}/dR_{1}=0$ and${d^{2}W/dR}_{1}^{2}$ = 0, yielding $\gamma_{l}=0.1055$ and $R_{1}^{*}/R_{1wo}^{*}=4/3$. Before reaching $\gamma_{l}$, a notable point, $\gamma_{n}$, can be identified where the nanodroplet and the vapor bubble have the same energy, indicating an equilibrium condition (*W =* 0). By solving $\mathrm{dW}/dR_{1}=0$ and W=0, the corresponding values $\gamma_{n}=1/16$ and ${R_{1}^{*}=2R}_{1wo}^{*}$ were obtained. Moreover, both the CR and the SCR disappeared when γ=0.2, signifying that under sufficient rigidity, the liquid nanodroplet became absolutely stable, even under negative pressure. In general, the nucleation process is energetically unfavorable, requiring a sufficient driving force, such as negative pressure, to overcome the energy barrier and trigger nucleation. However, in a sufficiently rigid medium, the external rigidity increases the energy barrier, further impeding nucleation. The nanodroplet is unable to deform or adjust to minimize the energetic cost of forming a new phase, resulting in the liquid phase remaining stable even under negative pressure. This phenomenon is consistent across liquid nanodroplets of varying sizes, with the primary distinction being the magnitude of the required energy. Larger nanodroplets experience lower internal pressure due to their smaller Laplace pressure, effectively reducing the energy barrier for bubble formation.

### 3.2 Nucleation rate at different ultrasonic pressures and temperatures

**Fig. 4:**
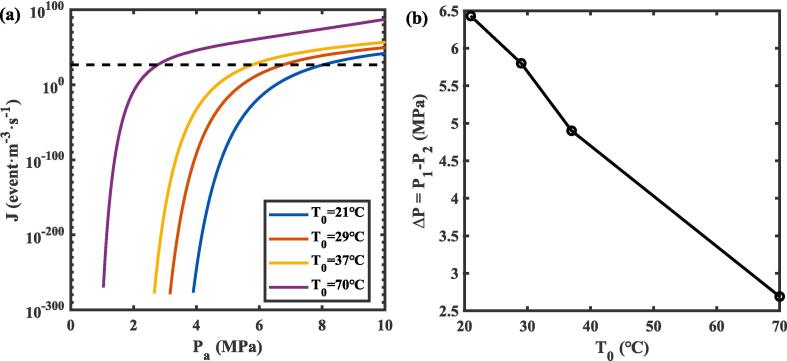
(a) Nucleation rate (*J*) as a function of ultrasonic pressure amplitude (*P*_a_) with temperature contours at *K*_3_ = 0.4 MPa. (b) The calculated under pressure (Δ*P*) for nucleation at different temperatures.

By solving the nucleation rate (Eq.12), the relationship between the nucleation rate and the ultrasonic pressure amplitude was determined for various temperature contours, as illustrated in Fig. 4 **(a)**. The nucleation rate J was expressed in units of vapor bubbles per cubic meter per second. J is approximated phenomenologically as $J=N_{b}/V_{20}\tau$ for steady-state nucleation [29], where $N_{b}$ is the number of vapor bubbles formed in the nanodroplet. For a given nanodroplet volume *V*_20_ and time interval *τ* = 1/10*f*, under the critical condition of forming the first vapor bubble (*N*_b_ = 1), the critical nucleation rate J* was approximately 4.4 × 10^26^, as shown in the dashed line in Fig. 4 **(a)**. This indicated that nucleation occurs when the temperature contours intersect with the J* threshold, nucleation rates below this threshold reflect insufficient conditions for vapor bubble formation. Once the first vapor bubble forms, the nanodroplet is not a single-phase liquid system. It should be noted that J was defined based on the typical phenomenological approach, and its value is more theoretical than practically applicable [29].

The nucleation rates were evaluated at different temperatures: *T*_0_ = 21 °C (sub-cooled state), 29 °C (boiling point), 37 °C (physiological temperature), and 70 °C (superheated state). Within the temperature interval of 21 °C–37 °C (away from the superheated state), the nucleation rate exhibited a modest increase as the ultrasonic pressure amplitude rose beyond the critical value J*. In contrast, for temperatures above 70 °C (superheated state), the nucleation rate increased dramatically by 74 orders of magnitude over the same pressure amplitude range after surpassing J*. This behavior indicates that ultrasonic pressure plays a more significant role in inducing vapor bubble nucleation at lower temperatures or in states far from superheating [42]. At higher temperatures, both ultrasonic pressure and temperature become key drivers of nucleation, with the process showing greater sensitivity to temperature variations [29].

As shown in Fig. 4 **(b)**, the under pressure (tension), defined as $\Delta P=P_{1}{-P}_{2}$, decreased with increasing temperature, while its influence gradually diminished with increasing temperature. Under conditions far from the thermodynamic limit of superheating, due to $P_{1}\gg P_{2}$ nucleation requires relatively higher pressures, and the temperature dependence of nucleation, as described by the exponent in Eq. (12), becomes less pronounced. However, as the temperature approaches the thermodynamic limit of liquid superheating, nucleation rates increase dramatically by several orders of magnitude per temperature increment. At these elevated temperatures, heating effects significantly influence the nucleation process and must be accounted for [43]. Due to the extremely short timescale of steady-state nucleation in nanodroplets—on the order of nanoseconds—it is challenging to achieve an effective temperature rise [16]. Therefore, this study focuses primarily on nucleation induced by ultrasonic pressure.

### 3.3 The initial nucleation threshold of a nanodroplet in tissue

#### 3.3.1 Effect of bulk modulus of tissues

**Fig. 5:**
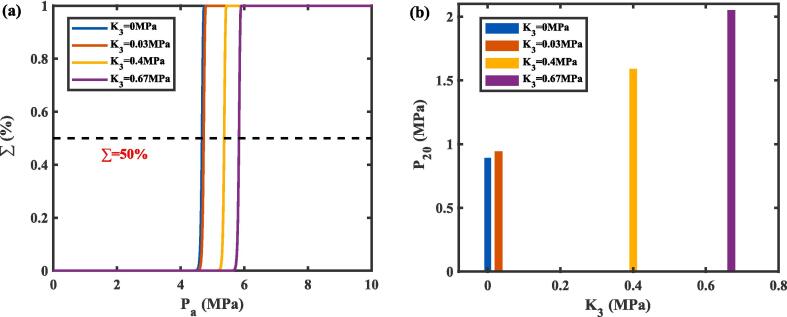
(a) Nucleation probability (∑) as a function of ultrasonic pressure amplitude (*P*_a_) under different tissue bulk moduli (*K*_3_). (b) Initial liquid pressures (*P*_20_) within the nanodroplet, calculated for tissue bulk moduli of *K*_3_ = 0 MPa, *K*_3_ = 0.03 MPa, *K*_3_ = 0.4 MPa, and *K*_3_ = 0.67 MPa at 37 °C.

Fig. 5**(a)** illustrates the nucleation probability (∑) as a function of the ultrasonic pressure amplitude (*P*_a_) for various tissue bulk moduli (*K*_3_). For a uniform and isotropic tissue following the laws of linear elasticity, *K*_3_ = 4*G*/3 [31], where *G* is the tissue shear modulus. In this study, four shear modulus cases were considered: *G* = 0 MP a (no shear modulus), *G* = 0.023 MPa (a low shear modulus, equivalent to a liver tissue phantom [44]), *G* = 0.3 MPa (a medium shear modulus), *G* = 0.43 MPa (a high shear modulus, equivalent to a kidney tissue phantom [44]). The corresponding bulk moduli were *K_3_* = 0 MPa, *K_3_* = 0.03 MPa, *K_3_* = 0.4 MPa, and *K_3_* = 0.67 MPa, respectively. The results indicated a significant dependence of the required ultrasonic pressure for nucleation on tissue stiffness. Specifically, as the bulk modulus increased, the pressure required to achieve a 50 % nucleation probability also increased. For example, the required pressure increased by 1.1 MPa as the bulk modulus changed from 0.03 MPa to 0.67 MPa. This trend aligns with the findings of Williams et al., who reported a similar correlation between vaporization threshold and phantom stiffness, where stiffer phantoms (with higher Young’s modulus) required higher ultrasonic pressures to initiate vaporization [27]. These observations suggest that tissue elasticity plays a critical role in inhibiting nanodroplet nucleation, as stiffer tissues impose greater mechanical resistance, necessitating higher pressures to overcome. However, only a 0.04 MPa rise in required ultrasonic pressure was observed when the bulk modulus ranged from 0 MPa to 0.03 MPa, indicating that within certain ranges of tissue stiffness, the impact on nucleation is minimal. This finding is consistent with studies on fibrin hydrogels, which showed that elasticity changes within a range (0.2 kPa to 12 kPa) had little effect on the vaporization threshold [19].

As the nanodroplet undergoes nucleation and vaporization, it expands and displaces the surrounding liquid-phase PFP, leading to the deformation of adjacent tissue. In comparison to the nucleation of nanodroplets in water, as modeled by Qin *et al*. [25], where the bulk modulus is effectively 0 MPa, the nucleated vapor bubble can freely displace the surrounding fluid with minimal resistance, requiring relatively low ultrasonic pressure for nucleation, as reflected in the modified CNT with a bulk modulus of 0 MPa. In tissues, however, the mechanical resistance imposed by elasticity significantly inhibits the nucleation process. As shown in Fig. 5**(b),** higher bulk moduli increase the initial pressure (*P*_20_) in the liquid nanodroplet, making the nanodroplet more stable and resistant to nucleation. Therefore, the constraining effect of tissue elasticity might prevent the vapor bubble nucleation rate from reaching the critical value required to vaporize the entire nanodroplet. This suggests that the initial vapor bubble may not be nucleated or vaporized if the critical nucleation rate is not reached due to tissue elasticity. Moreover, according to Eq. (5), the liquid pressures at the moment of nucleation at 37 °C were calculated for three different tissue bulk moduli, yielding values of approximately − 3.77 MPa. Notably, these liquid pressures predicted by the modified CNT were greater than the corresponding spinodal pressure at 37 °C, *P*_spin_ (−5.26 MPa), affirming the validity of the predicted INT. This distinction is crucial, as spinodal decomposition occurs spontaneously when the liquid pressure drops to *P*_spin_, enabling nucleation without the need for an external driving force [40].

#### 3.3.2 Effect of the presence of non-condensable dissolved gases in a nanodroplet

**Fig. 6:**
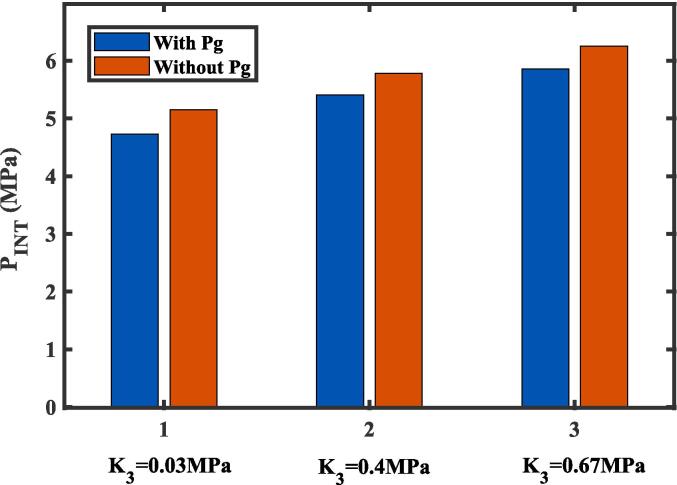
The effect of non-condensable dissolved gas on the initial nucleation threshold (P_INT_) of the nanodroplet in tissue at 37 °C.

Numerous researches have demonstrated that the amount of dissolved gas in a liquid significantly influences the initiation nucleation events [33], [45]. Liquid PFP exhibits a distinct advantage due to its exceptional capacity to dissolve gases such as O_2_ and CO_2_. For instance, under standard atmospheric pressure, the solubility of O_2_ in liquid PFP is approximately 20 times higher than that in water [34]. According to Eq. (9), the partial pressures of non-condensable dissolved gases can be determined by Henry’s law. The effect of dissolved gases on the INT of a nanodroplet in tissue was calculated for low, medium, and high tissue bulk moduli at 37 °C, as illustrated in Fig. 6. The partial pressures of non-condensable dissolved gases reduced the INT. The dissolved gases increase the vapor pressure inside the vapor bubble, thereby facilitating nucleation. This observation aligns with the measurements reported by Aliabouzar *et al*. [26]. After accounting for the partial pressure of non-condensable gases, Aliabouza *et al.* reported a reduction in nucleation pressure by approximately 2.2 MPa, whereas the reduction predicted by the modified CNT in this study was smaller. This discrepancy likely arises from differences in surface tension correction methods, as any changes in surface tension significantly impact nucleation threshold pressure. Aliabouza *et al*. considered the effect of dissolved gas content on liquid surface tension, which substantially reduces surface tension and further lowers nucleation pressure. Undoubtedly, the threshold pressure decreases as the dissolved gas concentration increases, highlighting the necessity of accounting for dissolved gases when calculating the total pressure inside the vapor bubble in systems with high gas solubility. While sufficient O_2_ is necessary for therapeutic effects in tumor tissues, the overall solubility of gases in solvents remains low. This means that the solutions typically fall within the range of dilute solutions, allowing Henry’s law to be applicable for calculating partial pressures within the vapor bubble. Although other dissolved gases, such as N_2_ and CO, are also present in liquid PFP, their contents are negligible compared with O_2_ and CO_2_ within the range studied here [34].

#### 3.3.3 Effect of nanodroplet properties

**Fig. 7:**
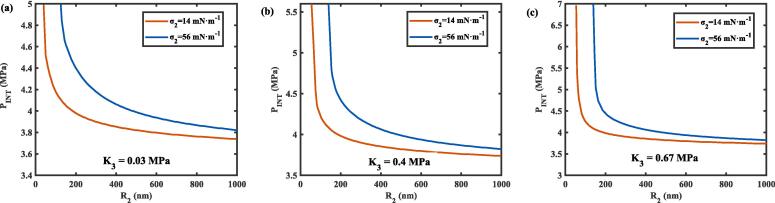
The effect of nanodroplet radius (*R*_2_) on the initial nucleation threshold (P_INT_) of the nanodroplet under different surface tension at the liquid-tissue interface (*σ*_2_) and bulk modulus of the tissue for (a) *K*_3_ = 0.03 MPa, (b) *K*_3_ = 0.4 MPa, and (c) *K*_3_ = 0.67 MPa at 37 °C.

The INT of a nanodroplet in tissue as a function of nanodroplet radius (*R*_2_) under different surface tension at the liquid-tissue interface (*σ*_2_) and tissue bulk moduli (*K*_3_) were illustrated in Fig. 7 **(a) − (c)**. The surface tension *σ*_2_ for uncoated PFP nanodroplets is 56 mN m^−1^ [46], while for coated lipids or fluorinated surfactants, it is reduced to 14 mN m^−1^ [47]. As the nanodroplet radius increased, the INT exhibited a decreasing trend across all conditions of bulk moduli and surface tension. Specifically, the INT decreased by approximately 0.5–3 MPa as the radius increased from 50 nm to 150 nm, and by 0.1–0.5 MPa as the radius increased from 150 nm to 1000 nm. This trend was consistent with the results of investigating the effect of droplet size on the vaporization threshold using both high-speed optical imaging and acoustic methods [48], [49]. Moreover, the curves corresponding to tissue with a bulk modulus of *K*_3_ = 0.67 MPa exhibiting steeper slopes compared to those with *K*_3_ = 0.4 MPa and *K*_3_ = 0.03 MPa, particularly in the range of smaller nanodroplets ($R_{2}$ < 150 nm). This indicated that the sensitivity of the INT to tissue elasticity is more pronounced in smaller nanodroplets and becomes increasingly evident as tissue elasticity increases. This may be because of the inversely proportional relation between the Laplace pressure and the nanodroplet radius, resulting in smaller nanodroplets exhibit higher initial liquid pressure (*P*_20_), making them more stable against nucleation, and a higher tissue bulk modulus further enhances the confinement effect. Encapsulating nanodroplets with lipids or fluorinated surfactants significantly reduces their INT while stabilizing them against coalescence, offering a practical strategy to achieve an optimal balance between in *vivo* stability and vaporization threshold.

In addition, an experimental nucleation threshold of 3.5 MPa was reported for lipid-coated nanodroplets with a mean diameter of 400 nm, exposed to 5 MHz ultrasound at 37 °C. This threshold was determined using optical methods and extrapolated from measurements of individual micrometer-sized droplets [50]. In contrast, our modified CNT model predicted an INT of 4.1 MPa for a lipid-coated nanodroplet with a radius of 200 nm, embedded in tissue with a bulk modulus of 0.02 MPa, and exposed to 5 MHz focused ultrasound at 37 °C. The slightly higher predicted value could be attributed to the limited sensitivity of acoustic-based experimental techniques for detecting nucleation thresholds in nanoscale droplets [51]. Furthermore, the high pressures required for nucleation may increase the likelihood of nanodroplet coalescence and radiation interactions in concentrated samples [52], potentially accounting for the lower experimental thresholds observed. However, the trend that the vaporization threshold decreases with the increase of nanodroplet size was consistent in the experiments.

#### 3.3.4 Effect of ultrasonic frequency

**Fig. 8:**
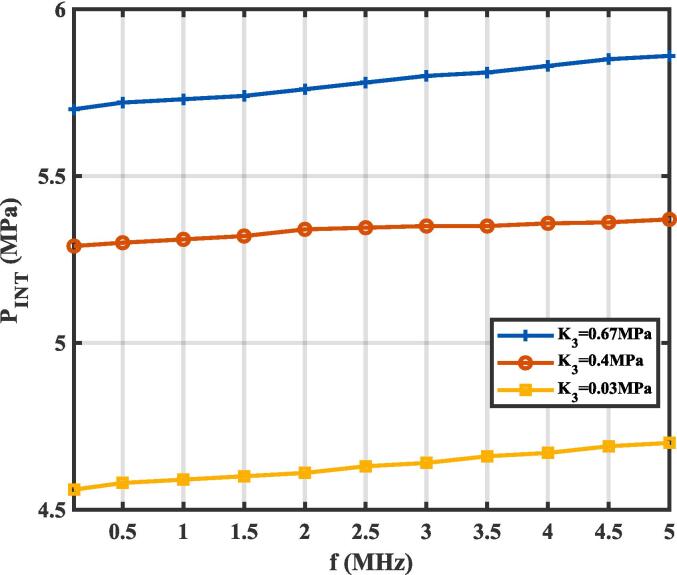
The effect of ultrasonic frequency (*f*) on the initial nucleation threshold of (P_INT_) the nanodroplet under tissue bulk moduli with *K*_3_ = 0.03 MPa, *K*_3_ = 0.4 MPa, and *K*_3_ = 0.67 MPa at 37 °C.

For steady nucleation, the dependence of the INT of a nanodroplet in tissue on the ultrasonic frequency was described by τ=1/10f. The effect of ultrasonic frequency (*f*) from 0.1 MHz to 5 MHz on the INT under tissue bulk moduli of *K*_3_ = 0.03 MPa, 0.4 MPa, and 0.67 MPa, is shown in Fig. 8. An increase in ultrasonic frequency resulted in a slight increase in the INT of a nanodroplet across all bulk moduli. Specifically, the INT increased by approximately 0.4 MPa when comparing 0.1 MHz and 5 MHz for a tissue bulk modulus of *K*_3_ = 0.67 MPa. At higher ultrasonic frequencies, the reduced acoustic period (*τ*) during which the nanodroplet experiences negative pressure leads to an increase in the INT. This weak dependence of the nucleation threshold on the acoustic period has been observed in studies using CNT to describe PFP microdroplet nucleation in water or cavitation [25], [26], with similar trends confirmed by experiment methods [53], [49]. However, the experimentally measured vaporization threshold exhibits a more pronounced dependence on driving frequency compared to the nucleation threshold predicted by the modified CNT. One possible explanation for this discrepancy is that lower frequencies or longer pulses of ultrasonic wave provide an extended time window for vapor bubble growth via rectified diffusion, making vapor bubbles easier to detect experimentally. Additionally, more ultrasonic attenuation and a significantly smaller focal volume at higher frequencies for experimental measurement, reduce the probability of nucleation events, further complicating the experimental detection of the effect of driving frequency on the vaporization threshold, while these effects are not considered in the modified CNT. Interestingly, an opposite trend has been observed when recording acoustic emissions from nanodroplets before and after vaporization [27], [54]. In these cases, the vaporization threshold decreases with increasing excitation frequency, this trend appears inconsistent with CNT. This counterintuitive observation has been attributed to factors such as heterogeneous nucleation or droplet deformation [15], nonlinear propagation and superharmonic focusing [24], [55]. At higher frequencies, the generation of higher-order harmonics and amplified superharmonic pressures may enhance nucleation dynamics, providing a plausible explanation for this phenomenon.

### 3.4 The achievable nucleation areas in tissue

The peak negative pressure of focused ultrasound is known to vary significantly due to notable ultrasonic attenuation in the megahertz frequency range and nonlinear effects during propagation through tissue, accompanied by a specific spatial distribution around the geometrical focus [56]. Within this nucleation region near the geometrical focus, nanodroplets typically exhibit a polydisperse size distribution, and the INT is inversely correlated with nanodroplet size, as discussed in 3.3.3. Consequently, polydisperse-sized nanodroplets may display distinct nucleation characteristics, particularly in terms of their achievable nucleation areas, under focused ultrasound of varying frequencies.

For ultrasound-induced nucleation, the achievable nucleation areas were identified as regions where the peak negative pressure amplitude exceeded the predicted INT. These peak negative pressure spatial distributions were generated using a single-element focused transducer with a radius of curvature of 60 mm, propagating through a two-layered medium (water + tissue). The distributions were calculated by solving the full wave nonlinear form of Westervelt equation using the open-source MATLAB toolbox, *k*-Wave Version 1.4 [57]. Detailed simulation parameters are provided in the supplementary materials. Given the widespread use of lipid-stabilized nanodroplets in theranostic applications targeting tumor tissue [58], [59], the INT calculations were conducted under the following conditions: a surface tension $\left(\sigma_{2}\right)$ of 14 mN m^−1^, a tissue bulk modulus (*K*_3_) of 0.03 MPa (representative of liver tissue), a temperature of 37 °C, and an ultrasonic frequency range from 1 MHz to 5 MHz.

**Fig. 9:**
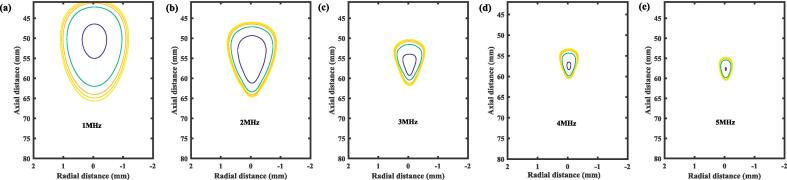
Achievable nucleation areas of lipid-coated nanodroplets ($\sigma_{2}$ = 14 mN m^−1^) in tissue (bulk modulus, *K*_3_ = 0.03 MPa) at 37 °C, under different frequencies. Contour lines (inner to outer) represent typical polydisperse nanodroplet diameters: 100 nm, 200 nm, 300 nm, 400 nm, and 500 nm. These contours represent the lower size limit that nanodroplets must achieve to initiate nucleation.

As shown in Fig. 9 **(a)-(e)**, the five contour lines in each subplot correspond to nanodroplet diameters ranging from 100 nm to 500 nm, increasing in 100 nm increments. These contours represented the lower size limit that nanodroplets must achieve to initiate nucleation. Such sizes enable nanodroplets to diffuse across the leaky tumor vasculature and preferentially accumulate in the tumor. Since the INT is inversely correlated with nanodroplet size (as discussed in Section 3.3.3) and the peak negative pressure amplitude was higher closer to the inner focus area, the innermost contour corresponds to the smallest nanodroplet size of 100 nm, while the outermost contour represents the largest nanodroplet size of 500 nm. This indicatesd that larger nanodroplets have an extender nucleation area. Furthermore, the achievable nucleation area increased significantly as the frequency decreased due to the attenuation effect at higher frequencies. For instance, at 1 MHz, the nucleation area was obviously larger than those at 5 MHz. Specifically, the length and width of the nucleation area for nanodroplets with a diameter of 500 nm differ by approximately 18.7 mm and 1.72 mm between 1 MHz (Fig. 9**(a)**) and 5 MHz (Fig. 9**(e)**), respectively. These results align with experimentally observed trends indicating that nucleation is more effective and covers larger areas at lower frequencies [60].

In addition, the length of the achievable nucleation area consistently exceeded its width across all frequencies. This phenomenon can be attributed to the geometric focus being predominantly distributed along the axial direction rather than the radial direction. The radial extension of the achievable nucleation area is influenced by thermal effects. At 1 MHz, the achievable nucleation area exhibited a relatively round shape. However, as the frequency increased, the nucleation area became more elongated, indicating an increase in the ratio of the length to the width of the nucleation area. Such trends are important in selecting an appropriate method for controlling and planning the activation of nanodroplets along the propagation direction, rather than the vertical plane. Notably, the nucleation area with the largest possible nucleation occurred pre-focally (57 mm) at all frequencies. In contrast, preferential nucleation generally took place around the geometric focus at 60 mm in the propagation direction. This highlights the disparity between the sites of preferential nucleation and the smallest nanodroplet sizes. In other words, the nanodroplets that nucleated at the geometric focus tend to be larger, while smaller nanodroplets typically occur nucleated at pre-focally. This indicated that the geometric focus is a location where nanodroplets are more likely to coalesce with others of similar size, forming larger nanodroplets that may be mechanically stable under the ultrasonic field [29].

## 4 Discussion

Nucleation is inherently a stochastic process. The modified CNT assumes that the first vapor bubble in a submicron-sized PFP nanodroplet within tissue is generated *via* homogeneous nucleation, which is consistent with theoretical models for vapor bubble nucleation in water or high intensity focused ultrasound applications [25], [29]. Li *et al.* confirmed this assumption that vapor bubbles nucleate inside the droplet under nanosecond timescales and high negative pressures by high-speed microscopy, where heterogeneous nucleation is unlikely [16]. Shpak et al. presented the spatial information of nucleation events in so-called 2D nucleation maps, which shows that the nucleation points with few events near the interface of the liquid PFP, suggesting a physical description based on homogeneous nucleation theory [17]. However, in nanodroplets with double or multi-phase emulsion structures (e.g., water-in-PFC-in-water systems embedded in hydrogels for tissue regeneration [26]), heterogeneous nucleation becomes more probable due to the presence of multiple phases. In such cases, incorporating geometric factors related to interfacial properties into CNT would enhance its predictive accuracy, and correspond to a relatively lower nucleation pressure threshold.

Moreover, compared with previous theoretical models attempting to investigate droplet nucleation in water (*γ* = 0), introducing the effects of nanodroplet compressibility and tissue elasticity (*γ* ≠ 0) in the total free energy of the vapor bubble as a function of their size, $W(R_{1})$, the vapor bubble underwent limited growth, allowing the existence of a stable solution (SCR). This modification resolves the issue of infinite growth and allows for a more realistic representation of the nucleation process. Notably, where the vapor bubble is surrounded by relatively high bulk modulus mediums, $W(R_{1})$ did not exist an SCR (*γ* = 0.2 in Fig. 3). In such scenarios, nucleation becomes impossible, even under conditions of high nucleation rates or significant negative pressures, as the energetic contribution of tissue elasticity is unfavorable for vapor bubble formation. Consequently, the nanodroplet remains metastable, unable to transition into a new stable phase, resulting in what is referred to as a “super-stabilization phenomenon” [61]. In negative pressure-driven nucleation, while not an intrinsic property of the liquid phase, the nucleation behavior is strongly influenced by the compressibility of the medium surrounding the vapor bubble [62]. This effect arises from the interplay between surface tension and the deformability of both the nanodroplet and the surrounding tissue. For small-sized nanodroplets within a sufficiently rigid system, the required negative pressure for nucleation becomes excessively high, causing the liquid phase to stretch extensively to fill the surrounding tissue, ultimately collapsing the vapor bubble. This phenomenon has been observed in several studies. For instance, Williams *et al.* observed no vaporization events in stiffer phantoms containing 200-nm PFH nanodroplets, even at the highest applied pressures [27]. These results demonstrate that under extreme confinement, homogeneous nucleation can be effectively arrested due to overstretching. This provides a means to control the phase transformation process and investigate the properties of nanodroplets under strongly metastable conditions, such as determining the equation of state of metastable nanodroplets or locating the spinodal line.

It is worth mentioning that tissues generally exhibit viscoelasticity characteristics, while the modified CNT the modified CNT emphasized the elastic effects of the tissue in this study. To assess whether viscous effects play a significant role before introducing the complexities of a viscoelastic model, we use the Deborah number (De) to evaluate the relative importance of elastic and viscous contributions in nucleation. It is mathematically expressed as: De=λ/θ=η/Gθ, where η and G are the shear viscosity and modulus, respectively, and θ is the time scale of the nucleation process. At the physiological temperature of 37℃, the tissue shear viscosity and modulus are approximately 10^-3^ Pa⋅s and 10^4^ Pa [63], respectively, and the nucleation time scale is about nanoseconds [51]. Thus, the Deborah number approaching 1 from values greater than 1, indicates that elastic effects dominate over viscous effects during nucleation process. Previous studies support this conclusion. For example, higher media viscosity, as indicated by increased fibrin density, was found to reduce the diameter of post-vaporization microbubbles by 2.5 % to 35 %, depending on fibrin concentration [19]. This highlights the significant role of viscosity in post-vaporization dynamics rather than in nanosecond-scale nucleation. Rojas et al. demonstrated a strong negative correlation between boundary constraints, imposed by tube size, and vaporization thresholds. While viscosity effects became more significant in smaller tubes (30 μm), they were minimal in larger ones (160 μm) [64]. These findings suggest that the boundary constraints imposed by the tube significantly influence the nucleation and vaporization, while viscosity becomes impactful only under high confinement. The boundary constraints in their experiments can be approximated as tissue-like elastic constraints, implying that when tissue elasticity is sufficiently high, viscosity should also be considered, as it may play a more critical role. While our current model does not include viscosity for simplicity, focusing on elasticity as the primary constraint, this approach is reasonable within the context of non-full-rigidness confinement. Nonetheless, the complex environment of the human body, where tissues may exhibit greater viscosity and elasticity, likely involves a synergistic interplay of these properties. Future research will explore the combined effects of viscosity and elasticity to better understand their influence on nanodroplet vaporization and be more aligned with the physiological conditions.

Previous work has demonstrated an inverse relationship between the vaporization threshold and the stiffness of polyacrylamide gel phantoms. Experimentally, the vaporization threhold increased from 4.8 ± 0.4 MPa in low-stiffness phantoms to 5.2 ± 0.2 MPa in high-stiffness phantoms under 1 μs pulses. The inverse relationship agreed with the prediction of our theoretical approach (Fig. 6). It is important to note that direct comparisons are complicated by the differences in experimental setups, droplet properties, and measurement methods. However, our model’s prediction of an INT of 4.75 MPa for 280 nm nanodroplets in a low-stiffness tissue at 37 ℃ agrees with the experimentally measured threshold of 4.9 MPa for 221 ± 12 nm PFP droplets exposed to 1-ms bursts in a low-stiffness phantom at 37 ℃ [27]. The thresholds were also comparable to the pressures reported by Zhang et al. for vaporizing 260 nm PFP nanodroplets in albumin-acrylamide gel phantoms and to those required to vaporize 200 nm PFP droplets at frequency of 4–8 MHz [65]. The tissue elasticity significantly increased the INT of a nanodroplet in tissue, highlighting its important role in the nucleation process, which is crucial for advancing extravascular applications. On one hand, this emphasizes the necessity of incorporating the elasticity of the surrounding medium into the CNT. On the other hand, it offers a means for identifying underlying pathologies by inferring changes in tissue elasticity from variations in the required ultrasonic pressure. Dynamic changes in the mechanical properties of tissues are often indicative of pathology. For instance, clinical studies have reported that the elastic modulus of healthy human liver tissue is typically below 6 kPa, whereas that of cancerous liver tissue can reach up to 70 kPa [66]. Such differences emphasize the potential of leveraging ultrasonic nucleation thresholds as indirect markers for tissue elasticity variations. Additionally, the INT exhibited a more pronounced increase for smaller-sized nanodroplets in higher bulk moduli (0.67 MPa), with a steeper change in behavior compared to larger nanodroplets (Fig. 7). This indicated that smaller nanodroplets are more sensitive to variations in tissue elasticity, whereas larger nanodroplets show diminished sensitivity. This result was consistent with previous experimental observations, which revealed that higher volume fractions of PFP droplets or longer pulse repetition frequency (PRF) increased the possibility of nanodroplet coalescence, leading to larger droplets and reducing the sensitivity of vaporization to the viscoelastic properties of the surrounding medium [19]. To maximize the sensitivity of vapor bubble nucleation to tissue viscoelastic properties and achieve more accurate pathological indications, it is essential to minimize nanodroplet coalescence and clustering (reducing nanodroplet concentration and PRFs), thereby enabling more precise differentiation of tissue elasticity in diagnostic and therapeutic applications.

Decreases in frequency were shown to reduce the INT of the nanodroplet (Fig. 8), which is consistent with findings reported in several previous studies on PFP droplets at 37 ℃. According to homogenous nucleation theory, liquid under negative pressure is metastable, and vapor bubbles could nucleate and grow if sufficient pressure and time are provided. Lower frequencies reduce acoustic energy attenuation, allowing negative pressure to remain effective over greater propagation depth, thereby increasing the likelihood of reaching the nucleation threshold in deeper tissues. Moreover, the longer wavelength associated with lower frequencies creates larger focal volumes, exposing more nanodroplets within tumors to the required acoustic conditions, thus enhancing the nucleation probability. Nucleation is expected once the vapor bubble reaches its critical radius at body temperature. Upon nucleation, the vapor bubble continues to grow until complete vaporization, transforming into a microbubble. It is worth noting that although recondensation of PFCs during subsequent acoustic cycles has been reported, this phenomenon typically occurs in heavier compounds like PFH with boiling points above body temperature [49], or in media with shear moduli exceeding 400 MPa [11]. As a result of the prolonged negative pressure phase, lower frequencies allow after-vaporized microbubbles to expand to at least twice their equilibrium size before collapsing violently, generating shock waves and/or liquid jets that increase the likelihood of inertial cavitation. The enhanced cavitation effect at lower frequencies has been particularly advantageous in nanodroplet-mediated histotripsy [67], [68], where it improves treatment efficiency by lowering the pressure threshold required to form a cavitation bubble cloud and tissue ablation. In contrast, the acoustic cycle is shorter at higher frequencies, which reduces the duration of the negative pressure phase. This limits the time available for vapor bubble nucleation and growth, resulting in an increased nucleation threshold. Furthermore, higher frequencies focus acoustic energy into smaller, more localized focal volumes, offering greater spatial precision but reducing the number of nanodroplets exposed to the required nucleation conditions. Experimental studies have shown that higher frequencies can generate amplified superharmonic pressures [55], which may influence nucleation dynamics through nonlinear effects and enhance localized cavitation efficiency, especially for larger droplets. However, this localized focus and rapid attenuation reduce the penetration depth of higher-frequency ultrasound, making it less effective for applications in deeper tissues. Despite these limitations, the precise control offered by higher frequencies is advantageous for applications requiring targeted nucleation, such as in confined tumor regions or near sensitive structures.

Additionally, the achievable nucleation areas for polydisperse-sized nanodroplets in the same ultrasonic field and tissue exhibited a clear dependence on nanodroplet size. Specifically, nanodroplets with a diameter of 100 nm showed significantly smaller nucleation areas compared to those sized between 200 and 500 nm, particularly under higher frequencies, such as 5 MHz. In experimental studies, the response of nanodroplets to ultrasound is often characterized using acoustic methods (detecting acoustic emissions before and after vaporization through active or passive cavitation detection techniques) [69]. Our findings suggested that these acoustic signals may predominantly originate from larger nanodroplets within the total volume of exposed PFP liquid, as larger droplets exhibit significantly increased nucleation areas. This phenomenon is consistent with the higher possibility of nucleation in larger nanodroplets. However, the existence of large nanodroplets during experimental characterization can introduce significant inaccuracies. Since nucleation areas increase with increasing nanodroplet size, resulting in larger nanodroplets may dominate the acoustic response, thereby distorting the representation of the entire nanodroplet population. To accurately characterize the nucleation process and avoid biases in acoustic measurements, it is essential to minimize the presence of large nanodroplets in experimental setups.

Previous studies have demonstrated that while ultrasonic pressure serves as the final trigger for nucleation, the achievable nucleation areas were often preferentially in regions of highest thermal deposition rather than at areas with peak negative pressure [29]. This observation seems inconsistent with our findings, where nucleation predominantly occurred pre-focally (Fig. 9**)**. However, this discrepancy can be explained by the inverse relationship between nucleation threshold and temperature. A significant rise in local temperature dramatically reduces the nucleation threshold. As a result, when the peak negative pressure exceeds this lowered threshold, thermal deposition can promote vapor bubble nucleation, aligning the nucleation regions with areas of the highest temperature. This observation underscores the critical role of thermal effects in the nucleation process, particularly under conditions where significant temperature rises occur. However, this study primarily focuses on nucleation at physiological temperatures, emphasizing the role of tissue mechanical properties. Incorporating temperature effects into the modified CNT to better capture the combined thermal and mechanical influences on nucleation is a promising direction for future investigations.

## 5 Conclusions

In this work, a modified CNT was developed to investigate vapor bubble nucleation occurring in a PFP nanodroplet in tissue. The results revealed that incorporating nanodroplet compressibility and tissue elasticity into the CNT significantly impacts the critical radius of the vapor bubble, highlighting the importance of considering these factors in modeling nucleation phenomena. Moreover, at higher temperatures (e.g., 70 °C), temperature dominates the nucleation process, whereas ultrasonic pressure plays a more prominent role at physiological and lower temperatures (25 °C–37 °C). Further analysis of the INT of a nanodroplet in tissue demonstrated that tissue bulk modulus has a significant impact on nucleation, with the INT increasing significantly as tissue elasticity increases. This highlighted the strong dependence of nucleation on the properties of the surrounding medium, offering the potential for identifying pathologies such as fibrosis and cancer. Notably, smaller nanodroplets were found to be more sensitive to changes in tissue elasticity, suggesting that reducing nanodroplet size could enhance the accuracy of pathological assessments. The presence of dissolved gases, increasing nanodroplet surface tension, and decreasing nanodroplet radius and ultrasound frequency reduced the INT, providing a practical strategy for optimal nucleation parameters. Additionally, the study revealed that achievable nucleation areas expanded as ultrasonic frequency decreased. The length of the nucleation region was consistently greater than its width, presenting opportunities for controlled and directional nanodroplet vaporization along the propagation axis. This anisotropic behavior could be leveraged for precise targeting in theranostic applications. Overall, the results of this study provide valuable insights into the role of various parameters in the vapor bubble nucleation in metastable PFP nanodroplets in tissue and contribute to a deeper understanding of nanodroplet nucleation in tissues.

## CRediT authorship contribution statement

**Kangyi Feng:** Writing – review & editing, Writing – original draft, Validation, Software, Methodology, Investigation, Formal analysis, Data curation, Conceptualization. **Yueyuan Wang:** Writing – review & editing, Validation, Software. **Chaonan Zhang:** Writing – review & editing, Software. **Anqi Huang:** Formal analysis, Conceptualization. **Mingxi Wan:** Writing – review & editing, Supervision, Resources, Investigation, Funding acquisition. **Yujin Zong:** Writing – review & editing, Supervision, Methodology, Investigation, Funding acquisition, Formal analysis, Conceptualization.

## Declaration of competing interest

The authors declare that they have no known competing financial interests or personal relationships that could have appeared to influence the work reported in this paper.
