# Supplementary material for: Initial nucleation of nanodroplets in viscoelastic tissue driven by ultrasound: A theoretical simulation
Source: Ultrason Sonochem. 2025 Feb 24;115:107285. doi: 10.1016/j.ultsonch.2025.107285 (PMC11926720; doi:10.1016/j.ultsonch.2025.107285)
Supplement: Supplementary Data 1 [file mmc1.docx]

***Supplementary Information***

**Initial Nucleation of Nanodroplets in Viscoelastic Tissue Driven by Ultrasound: A Theoretical Simulation**

*Kangyi Feng, Yueyuan Wang, Chaonan Zhang, Anqi Huang, Mingxi Wan*, Yujin Zong**

*The Key Laboratory of Biomedical Information Engineering of Ministry of Education, School of Life Science and Technology, Xi’an Jiaotong University, Xi'an 710049, People's Republic of China*

* Corresponding author. E-mail: mxwan@mail.xjtu.edu.cn (M. Wan) & yjzong@mail.xjtu.edu.cn (Y. Zong)

Nonlinear acoustic propagation in a multi-layered tissue medium (i.e., water + tissue) was simulated for a single element-focused transducer (the curvature radius of 60 mm, a radius of 28.5 mm). In order to reduce the amount of calculation and improve computing efficiency, a 2D axisymmetric coordinate system was used instead of a 3D Cartesian coordinate system. The total size of the computational domain used was 80 mm×40 mm, which was divided into 2048 ×1024 grid points, including a perfectly matched layer (PML) of 20 × 20 grid points on each side of the domain. The Courant-Friedrichs-Lewy (CFL) number was set to 0.05, which means a temporal step size Δ*t* of 1.3 ns and a grid spacing (Δ*x* and Δ*y*) of 39.2 μm in the axial and lateral directions, and the maximum frequency supported in the simulation is 19.076 MHz. We selected five different ultrasonic frequencies, 1- 5 MHz with increment of 1 MHz. The physical properties used in the simulations were listed in Table 1.

Table 1 Physical parameters used in simulations.

|  | Water [1,2] | Tissue [3] |
| --- | --- | --- |
| Density (kg/m^3^) | 1000 | 1055 |
| Speed of sound (m/s) | 1520 | 1570 |
| α_0_ (Np/m) | 0.026 | 8.1 |
| Power law exponent | 2 | 1 |
| Coefficient of nonlinearity | 3.5 | 4.5 |
| Propagation distance (mm) | 40 | 40 |

**Reference**

[1] E. Martin, J. Jaros, B.E. Treeby, Experimental Validation of k-Wave: Nonlinear Wave Propagation in Layered, Absorbing Fluid Media, IEEE Trans. Ultrason. Ferroelectr. Freq. Control 67 (2020) 81–91. https://doi.org/10.1109/TUFFC.2019.2941795.

[2] J. Robertson, J. Urban, J. Stitzel, B.E. Treeby, The effects of image homogenisation on simulated transcranial ultrasound propagation, Phys. Med. Biol. 63 (2018) 145014. https://doi.org/10.1088/1361-6560/aacc33.

[3] P. Gupta, A. Srivastava, Numerical analysis of thermal response of tissues subjected to high intensity focused ultrasound, Int. J. Hyperthermia 35 (2018) 419–434. https://doi.org/10.1080/02656736.2018.1506166.
